# The peritumoral brain zone in glioblastoma: where we are and where we are going

**DOI:** 10.1002/jnr.25134

**Published:** 2022-10-27

**Authors:** Martina Giambra, Andrea Di Cristofori, Silvia Valtorta, Roberto Manfrellotti, Vittorio Bigiogera, Gianpaolo Basso, Rosa Maria Moresco, Carlo Giussani, Angela Bentivegna

**Affiliations:** ^1^ School of Medicine and Surgery University of Milano‐Bicocca Monza Italy; ^2^ PhD Program in Neuroscience University of Milano‐Bicocca Monza Italy; ^3^ Division of Neurosurgery Azienda Socio Sanitaria Territoriale – Monza, Ospedale San Gerardo Monza Italy; ^4^ Department of Nuclear Medicine San Raffaele Scientific Institute, IRCCS Milan Italy; ^5^ Institute of Molecular Bioimaging and Physiology National Research Council (IBFM‐CNR) Segrate Italy; ^6^ NBFC National Biodiversity Future Center 90133 Palermo Italy

**Keywords:** glioblastoma, peritumoral brain zone, multimodal imaging

## Abstract

Glioblastoma (GBM) is the most aggressive and invasive primary brain tumor. Current therapies are not curative, and patients' outcomes remain poor with an overall survival of 20.9 months after surgery. The typical growing pattern of GBM develops by infiltrating the surrounding apparent normal brain tissue within which the recurrence is expected to appear in the majority of cases. Thus, in the last decades, an increased interest has developed to investigate the cellular and molecular interactions between GBM and the peritumoral brain zone (PBZ) bordering the tumor tissue. The aim of this review is to provide up‐to‐date knowledge about the oncogenic properties of the PBZ to highlight possible druggable targets for more effective treatment of GBM by limiting the formation of recurrence, which is almost inevitable in the majority of patients. Starting from the description of the cellular components, passing through the illustration of the molecular profiles, we finally focused on more clinical aspects, represented by imaging and radiological details. The complete picture that emerges from this review could provide new input for future investigations aimed at identifying new effective strategies to eradicate this still incurable tumor.

**Significance**

Glioblastoma (GBM) is the most aggressive and invasive of the primary brain tumors. Despite surgical removal, followed by radiation and chemotherapy with temozolomide, to date GBM is still incurable, and the prognosis for patients is very poor with only half the patients surviving for 20.9 months after surgery. One of the main reason for the incurability of GBM is its high rate of recurrence, probably due to the inability to completely remove the tumor mass. GBM develops through infiltration into the apparently normal tissue bordering the tumor, the peritumoral brain zone (PBZ), which is the topic we focused on in this review.

## 1 INTRODUCTION

Glioblastoma (GBM) is the most aggressive and invasive of the primary brain tumors (Louis et al., 2021). Despite gross total surgical resection and adjuvant chemotherapy with the alkylating agent temozolomide (TMZ) and radiotherapy, patients with GBM have a poor median overall survival (OS) of 14–16 months (Ricard et al., 2012). Recently, with the introduction of the tumor‐treating fields approach, median OS has increased to 20.9 months after surgery (Fernandes et al., 2017; Stupp et al., 2017). Nevertheless, recurrence is expected in 90% of cases within 12 months after diagnosis (Ricard et al., 2012).

Cellular heterogeneity is a main feature of GBM and, among the different tumoral cell populations, the glioma stem cells (GSCs) are thought to contribute the most to tumor spreading, invasion, proliferation, and maintenance, seeming to be responsible for cancer chemo‐radio resistance (D'Alessio et al., 2019; Persano et al., 2011).

GBM is also characterized by molecular heterogeneity. To date, the new WHO 2021 classification (Louis et al., 2021) identifies chromosome 7 amplification and chromosome 10 deletion as the key hallmarks for GBM diagnosis. In 2010, Verhaak and colleagues proposed a more detailed molecular categorization of GBM into four subtypes: proneural, mesenchymal, classical, and neural. According to this, only the classical subtype would fit with the current WHO 2021's GBM definition, showing focal 9p21.3 homozygous deletions that target CDKN2A, in addition to chromosome 7 amplification and chromosome 10 deletion. Furthermore, classical GBM markers include Nestin, NOTCH3, JAG1, LFNG, SMO, GAS1, and GLI2. PDGFRA alterations and IDH1 mutations characterize the proneural subtype, along with higher expression of certain proneural markers (e.g., SOX, DCX, DLL3, ASCL1, and TCF4) and oligodendrocytic development genes (e.g., PDGFRA, NKX2‐2, and OLIG2). The mesenchymal subtype is featured by focal 17q11.2 homozygous deletions, co‐mutations in NF1 and PTEN genes, enrichment of TNF super family pathway and NF‐κB pathway genes (e.g., TRADD, RELB, and TNFRSF1A), and high expression of mesenchymal and astrocytic markers, such as CD44 or MERTK, which promote epithelial–mesenchymal transition (EMT). Lastly, the neural subtype is characterized by normal brain tissue gene expression profile as well as astrocytic/oligodendrocytic cell markers and expression of neuronal markers such as NEFL, GABRA1, SYT1, and SLC12A5 (Verhaak et al., 2010).

GBM develops through the infiltration into the apparently normal adjacent brain tissue within which the recurrence is expected to appear in the majority of cases (Ricard et al., 2012). Thus, in the last decades, an increased interest has developed to investigate the cellular and molecular interactions between GBM and the peritumoral brain zone (PBZ) bordering the tumor tissue. The aim of this review is to provide up‐to‐date knowledge about the possible pro‐oncogenic potential of the PBZ to highlight feasible druggable targets for more effective treatment of GBM to limit the formation of recurrence, which is almost inevitable in the majority of patients.

## 2 THE PERITUMORAL BRAIN ZONE: TOWARD AN UNAMBIGUOUS DEFINITION

To date there is no single definition of PBZ, probably due to a still very limited knowledge of this region. In this review, we refer to PBZ as the marginal area surrounding the tumor core (TC), but in the literature, the definition may be different from time to time. For instance, Lemèe and coworkers (Lemée, Clavreul, & Menei, 2015) define four regions of interest that, moving from the center of the tumor toward its surroundings, should be identified as the necrotic zone, the florid tumor zone, the interface zone (IZ), and the PBZ. Conversely, Laytysheva et al., through radiological imaging, define the PBZ as the area extended outside the contrast‐enhancing (CE) TC, composed by three different layers: the peri‐enhancing zone, extended 5 mm over the peritumoral CE ring; the near zone, between 5 and 10 mm; and the far zone, between 10 and 15 mm, from CE region (Latysheva et al., 2020). The pro‐oncogenic potential of the PBZ is supported by the possibility that recurrence happens mostly at the margins of the resection cavity (Lemée, Clavreul, Aubry, et al., 2015). Moreover, previous studies found variable cellular components in this area with different carcinogenic properties, probably responsible for tumor recurrence (Clavreul et al., 2015; Petrecca et al., 2013; Wang et al., 2015).

## 3 CELL HETEROGENEITY

**FIGURE 1:**
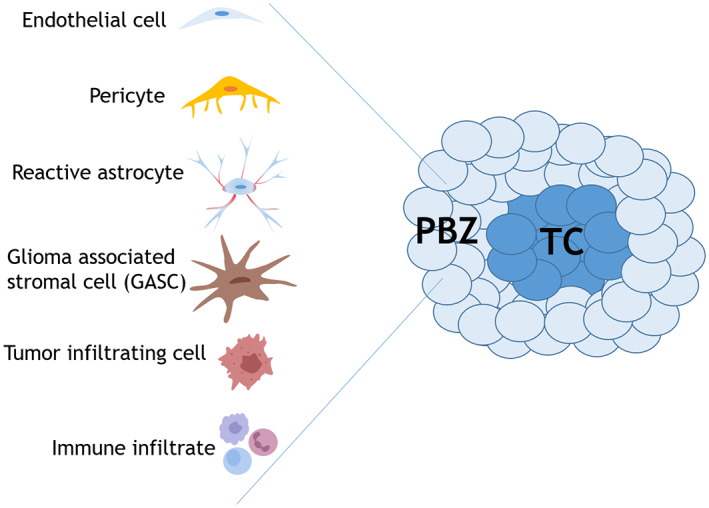
Schematic representation of the cellular heterogeneity in the peritumoral brain zone (PBZ) that surrounds the tumor core (TC) of high‐grade gliomas.

Variable cellular components have been identified within the PBZ area, but their role has not been fully understood yet (Figure 1).

### 3.1 Vascular cells

Endothelial cells (ECs) are CD34, CD31, and CD133 positive and express Tie2 and VE cadherin markers (Asahara et al., 1997). During their differentiation, endothelial progenitors begin to express VEGFR‐1/2, Tie1, and E selectin (Asahara et al., 1997; Miettinen et al., 2012). Tamura and coworkers observed that, within the TC of GBM, almost all CD31 and CD34‐positive ECs co‐expressed VEGFR‐1 and ‐2 (Tamura, Ohara, Sasaki, Morimoto, Yoshida, et al., 2018). Conversely, in the PBZ most CD31 and CD34‐positive ECs did not express VEGFRs, showing positivity to Nestin, and negativity to Factor VIII (Table 1). This latter consideration may indicate a process of tumor vessel formation in the PBZ, since Nestin is an endothelial progenitor marker, while Factor VIII is related to mature capillaries. Moreover, low expression of VEGFRs in ECs of the PBZ seems to correlate with the treatment failure of VEGFR‐targeting drugs, and it could explain why this area often represents the site of recurrence after surgical resection. Furthermore, the expression of VEGF‐A in the PBZ is lower than in the TC, but it is higher than that in TCs of lower‐grade gliomas (Tamura, Ohara, Sasaki, Morimoto, Kosugi, et al., 2018).

**TABLE 1:** Specific cellular phenotypes of the tumor core (TC) and of the peritumoral brain zone (PBZ) of high‐grade gliomas (Bastola et al., 2020; Clavreul et al., 2012, 2014; Rahimi Koshkaki et al., 2020; Tamura, Ohara, Sasaki, Morimoto, Kosugi, et al., 2018; Tamura, Ohara, Sasaki, Morimoto, Yoshida, et al., 2018)

Cell type	Phenotype in TC	Phenotype in PBZ
Endothelial cells	CD31+, CD34+, VEGFR1/2+	CD31+, CD34+, VEGFR1/2−, Nestin+, Factor VIII−
Immune infiltrate	HIF‐1a+, VEGF‐A+, Foxp3+ CD163+, CD8−, CD3+, PD‐1+, PDL‐1+, TIGIT+, IDO+, T‐cell exhaustion+	HIF‐1a−, VEGF‐A−, Foxp3− CD163−, CD8+, CD3−, PD‐1−, PDL‐1−, TIGIT−, IDO−, T‐cell exhaustion−
Tumor cells	CD109+, CD44+, MYC+, HIF1α+, VIM+, ANXA1+, CDK6+, JAG1+	OLIG2+, OLIG1+, TC2+, SRRM2+, ERBB3+, PHGDH+, RAP1GAP+
Glioma‐associated stromal cells	/	CD105+, CD73+, CD90+, CD44+, CD14−, CD34−, CD45−, αSMA+, PDGFRβ/CD140b+, FSP1/S100A4+

Perivascular cells, referred to as pericytes, contribute to vessel integrity and GBM neurobiology. In particular, they have a key role in regulating the blood–brain‐barrier permeability, in promoting angiogenesis, in aiding the degradation of the extracellular matrix (ECM), as well as in assisting the immune surveillance evasion, ultimately allowing tumor growth.

To date, a pathogenic crosstalk between tumor cells and pericytes has been proved through cytoplasmic extensions called “flectopodia.” This mechanism results in the co‐option of modified pre‐existing blood vessels that support the expansion of the disease toward the peritumoral area (Caspani et al., 2014). “Flectopodia” is the proper name of the specific interaction between tumor cells and pericytes via thin membranous tubes. This type of communications, made by actin prolongations, is known by several names such as tunneling nanotubes, tumor microtubes, membrane bridges, and they seem to have a key role in driving GBM therapy resistance (Taiarol et al., 2021).

It is well known that GBM is characterized by necrosis and microvascular hyperplasia. Necrotic foci are typically surrounded by “pseudopalisading” cells, which are severely hypoxic, overexpress hypoxia‐inducible factor (HIF‐1), and secrete proangiogenic factors such as VEGF and IL‐8. Thus, the microvascular hyperplasia in GBM that provides a new vasculature and promotes peripheral tumor expansion is tightly linked with the branch of pseudopalisades (Brat et al., 2004; Rong et al., 2006).

### 3.2 Immune infiltrate

GBM is widely recognized as an immunosuppressive disease. Immune cells include many kinds of myeloid cells such as tumor‐associated macrophages (TAMs), consisting in both brain‐resident microglia and bone marrow–derived macrophages, myeloid‐derived suppressor cells, dendritic cells, and neutrophils (Tamura, Ohara, Sasaki, Morimoto, Kosugi, et al., 2018). The most abundant non‐neoplastic cells are a population of CD163+ M2‐like TAMs, which support tumor proliferation, regulate immunosuppression, and contribute to cerebral edema (De Leo et al., 2020). A recent in vitro study by Gabrusiewicz and coworkers (2018) revealed that the phenotypic switch of CD14+ monocyte precursor toward M2‐like TAMs is due, at least in part, to GSC‐derived exosomes. These vesicles contain, among the others, members of the signal transducer and activator of transcription 3 (STAT3) pathway. The exosome‐activated monocytes go through cytoskeleton‐morphological changes; they increase phagocytosis, upregulate PD‐L1 and cytokines, such as MCP‐3 and CXCL1, and ultimately contribute to GBM immunosuppressive microenvironment in the PBZ (Gabrusiewicz et al., 2018). Tamura et al. carried out a detailed study on the immune compartment in the PBZ, highlighting differences with the TC. First of all, HIF‐1a and VEGF‐A are broadly expressed in TC, unlike the PBZ, where they are slightly expressed. Additionally, even the number of Foxp3+ and CD163+ cells is significantly lower in the PBZ than in the TC, where they are diffusely observed in the broad area, including the perivascular area. Conversely, the number of CD8+ T‐cells is significantly lower in the TC compared with the PBZ, while CD4+ T‐cells and PD‐1+ lymphocytes are widely present in both areas (Tamura, Ohara, Sasaki, Morimoto, Kosugi, et al., 2018) (Table 1). Koshkaki and colleagues tried to deepen the knowledge about the localization and distribution of the main immunological markers and targets for immunotherapies in both areas. They highlighted that CD163+ M2‐like TAMs and CD3+ T‐cells, are more relevant in the TC, showing a gradient from the periphery to the TC, where they accumulate (Rahimi Koshkaki et al., 2020) (Table 1). The closeness between these two cell populations may explain TAMs' immunosuppressive effect on T‐cells, which may account for the failure of all of the recent trials of immune checkpoint inhibitors. In the peripheral area, Koshkaki confirmed the strong reduction of the immunosuppressive marker FoxP3, already observed by Tamura, as well as of PD‐1, PDL‐1, TIGIT, and IDO (Rahimi Koshkaki et al., 2020; Tamura, Ohara, Sasaki, Morimoto, Kosugi, et al., 2018). Moreover, evidence reported that PBZ hosts more effector T‐cells that have not experienced exhaustion, and this would suggest a more suitable environment for the efficacy of immunotherapies (Tamura, Ohara, Sasaki, Morimoto, Kosugi, et al., 2018) (Table 1).

### 3.3 Reactive astrocytes

Astrocytes comprise approximately 50% of the cells in the brain. Traditionally they were thought to provide structural support, with additional roles in homeostasis and cellular communication. Activated astrocytes play a crucial role in the progression, aggressiveness, and angiogenic process of the tumor mass and have been observed both in the TC and in the margin (Placone et al., 2016). The astrocyte transition into a reactive cell status is called “reactive gliosis” and is promoted by activated microglia and tumor cells that secrete pro‐inflammatory cytokines, such as Il‐1α, TNF, and C1q (Birck et al., 2016; Liddelow et al., 2017). Reactive astrocytes express high levels of glial fibrillary acidic protein (GFAP) and support parenchymal infiltration and uncontrolled proliferation of glioma cells by expressing matrix metalloproteinase‐2 (MMP2) and secreting stromal cell–derived factor‐1 (SDF1), respectively (Barbero et al., 2002; Gagliano et al., 2009). Reactive astrocytes and cancer cells can directly communicate via gap junction channel protein connexin43 (Cx43) and by tunneling nanotubes, long and thin tubular structures established between the two types of cells. In addition, indirect communication is supported by reactive astrocyte secretion of chemokines or cytokines, such as interleukin‐6 (IL‐6), transforming growth factor‐ β (TGF‐β), insulin‐like growth factor‐1 (IGF‐1), monocyte chemotactic protein‐4 (MCP‐4), interleukin‐19 (IL‐19), vascular endothelial growth factor (VEGF), and leukemia inhibitory factor, among others, to promote tumor cell invasion and migration, proliferation, and growth (Guan et al., 2018). Recently, Niklasson and coworkers demonstrated that the mesenchymal GBM subtype and reactive astrocytes show very similar expression signature. Furthermore, through gene set enrichment analysis and RNA‐seq, they revealed a strong association between treatment resistance linked to GBM intratumoral heterogeneity and differentially expressed genes connected to astrocyte reactivity (Niklasson et al., 2019). Finally, CD44 ligand osteopontin secretion by astrocytes can enhance a stem cell–like phenotype and radiation resistance in glioma cells, while promoting GBM growth in vivo (Pietras et al., 2014).

### 3.4 Tumor infiltrating cells

GBMs are characterized by extensive and diffuse infiltration in the brain parenchyma. Glioma cells preferentially invade along myelinated fibers in white matter tracts (intrafascicular growth), in cerebrospinal fluid pathways or in meninges, and thus give rise to multifocal gliomas. This is possible thanks to the presence of intracellular systems that coordinate all incoming and outgoing signals, a locomotor apparatus in which the actin cytoskeleton plays a crucial role; the ECM on which the glioma cells can travel; molecules that remove obstacles, like ECM degrading proteases; growth factors that guide the way, and other stimulatory or permissive microenvironmental factors (e.g., chemokines derived from inflammatory cells) (Claes et al., 2007). Bastola and colleagues in a recent study firstly demonstrated that tumor cells from core and edge tissues exhibit distinct molecular properties: the former are enriched with Olig2+ cells, while the latter have a greater expression of CD109, a putative core marker. A substantial difference has been also highlighted: the TC expresses CD44, MYC, HIF1α, VIM, ANXA1, CDK6, JAG1, whereas the PBZ expresses OLIG1, TC2, SRRM2, ERBB3, PHGDH, and RAP1GAP (Table 1). The same authors also demonstrated that intercellular signaling from GBM core cells promotes growth and increases radiation resistance of PBZ cells via HDAC1‐CD109 dependent manner (Bastola et al., 2020). Furthermore, PBZ areas of different subtypes show differences in specific markers: the proneural margins contain mainly Olig2+/OPC‐like cells, which probably represent infiltrating glioma cells; the mesenchymal margins are enriched with genes expressed by microglia and CD44+ astrocytes; the classical margins contain a mixture of Olig2+ and CD44+ glioma cells (Gill et al., 2014) (Table 2). Nimbalkar et al. found that SERPINA3 expression was higher in PBZ than in the TC (see paragraph 4.2), and its knockdown in vitro decreased tumor cell proliferation, invasion, migration, transition to mesenchymal phenotype, stemness, and radio resistance (Nimbalkar et al., 2021). A new studied mechanism in GBM recurrence is the formation of a particular type of membrane tubes that facilitate intercellular communication, termed tumor microtubes. They are longer and have greater diameters in comparison with tunneling nanotubes observed in vitro (Roehlecke & Schmidt, 2020). Weil et al. through multiphoton laser scanning microscopy in animal models observed that tumor cells left over in the surgical resection margins extended new tumor microtubes toward and then into the lesioned area, causing tumor cells recolonization. These data suggest that tumor microtubes may be used as drug target to fight resistance and recurrence in glioma (Taiarol et al., 2021; Weil et al., 2017).

**TABLE 2:** Cellular components identified in the peritumoral margin (PBZ) of different glioblastoma (GBM) subtypes (Gill et al., 2014)

GBM molecular subtype	Specific cells in PBZ
Proneuronal	Olig2+/OPC‐like cells
Mesenchymal	microglia and CD44+ astrocytes
Classical	mixture of Olig2+ and CD44+ glioma cells

### 3.5 Glioma‐associated stromal cells

Recent studies reported that GBM environment harbors cells with characteristics of mesenchymal stem cells (MSCs) and cancer‐associated fibroblasts (CAFs), frequently described as “glioma‐associated stromal cells” (GASCs) (Clavreul & Menei, 2020). GASCs express classical MSC surface markers, such as CD105, CD73, CD90, and CD44, and lack the expression of CD14, CD34, and CD45; they also express markers associated with CAFs, including alpha‐smooth muscle actin (αSMA), platelet‐derived growth factor receptor‐beta (PDGFRβ/CD140b) and FSP1/S100A4 (Clavreul et al., 2012, 2014) (Table 1). Several hypotheses attribute their origin to a transdifferentiation of pericytes and vascular smooth muscles cells or to EMT of ECs; they may also originate from MSCs that have tropism for gliomas (Clavreul & Menei, 2020; Gomes et al., 2018; Yoshida, 2020). In addition, a possible malignant transformation of MSCs after interacting with GSCs could be associated with the downregulation of miR‐146a‐5p that enhances the overexpression of the oncogene p37^AUF1^ (HNRNPD) (Dai et al., 2020), already seen to be involved in the EMT in human mammary epithelial cells (AlAhmari et al., 2020). Albeit their origin from GSCs has been hypothesized, since GASCs are diploid and do not present the canonical GSC alterations, this hypothesis is not widely considered (Clavreul et al., 2012). Despite the controversial origin, these cells have been shown to play an active role in reciprocal communication with tumor cells, thereby accelerating tumor growth and progression, and to have angiogenic properties (Clavreul et al., 2012, 2014). Zhang and coworkers investigated the role of growth factors, such as PDGF‐BB and TGF‐β1 in GASCs. They demonstrated that TGF‐β1 receptor and PDGF receptor are expressed in GASCs and that their expression is increased during angiogenesis in response to TGF‐ β1 and PDGF‐BB, respectively, suggesting that these mechanisms may provide new insights into the possibility of targeting vessel formation in PBZ as well (Zhang et al., 2021). GASCs seem to stimulate migration and invasion mechanisms in tumor cells through soluble factors secretions, including growth‐regulated oncogene alpha (GROα), interleukin (IL)‐6, IL‐8, and above all C5a, which through MAPK signaling, increases the level of the oncogene ZEB1 (Lim et al., 2020). For instance, a recent report by Figueroa J. and colleagues showed that extracellular vesicles (EVs) secreted from GASCs are a source of growth factors and morphogens, which contribute to GSC self‐renewal and tumor growth. In particular, these effects are associated with miR‐1587 transfer via exosomes in GSCs. This miRNA downregulates the tumor‐suppressive nuclear receptor co‐repressor NCOR1, developing a greater tumor volume and causing decreased survival in orthotopic xenografts (Figueroa et al., 2017). However, the role of stromal cells in GBM is still unclear because there are conflicting studies on whether they promote or suppress GBM growth, proliferation, and migration. For instance, studying the mRNA content of EVs released by GASCs, Xu and colleagues found out that miR133b has a tumor‐suppressive effect on glioma development, through inhibition of the EZH2 and Wnt/β‐catenin signaling pathway (Xu et al., 2019). Similarly, Ho and coworkers observed a reduced proliferation of glioma cells in GASC/glioma co‐cultures and a lower expression of PDGF‐BB and IL‐1β compared with controls. Furthermore, a decrease in tumor volume and vascular density was detected in animal models injected with GASCs and glioma cells. These data validated the hypothesis that stromal cells have an antitumor effect suppressing cancer angiogenesis (Ho et al., 2013). On the contrary, in other studies the presence of GASCs is associated with a poor prognosis. Indeed, their presence has been demonstrated to be negatively correlated with good outcomes in GBM patients, particularly the high percentage of these cells, in patients' tumor bulks or cell cultures, is associated with a poor OS (Clavreul & Menei, 2020; He et al., 2018; Shahar et al., 2017; Yoon et al., 2016).

## 4 MOLECULAR HETEROGENEITY

Multiple methods have been developed to classify tumors according to the key molecular events that drive the most aggressive cellular components so that targeted therapies can be developed for individual subtypes. However, targeted or tailored therapies for specific mutations or subtypes largely failed due to the tumor complexity arising from intra‐tumoral molecular heterogeneity (Lee et al., 2018).

### 4.1 Genomic profiles

**FIGURE 2:**
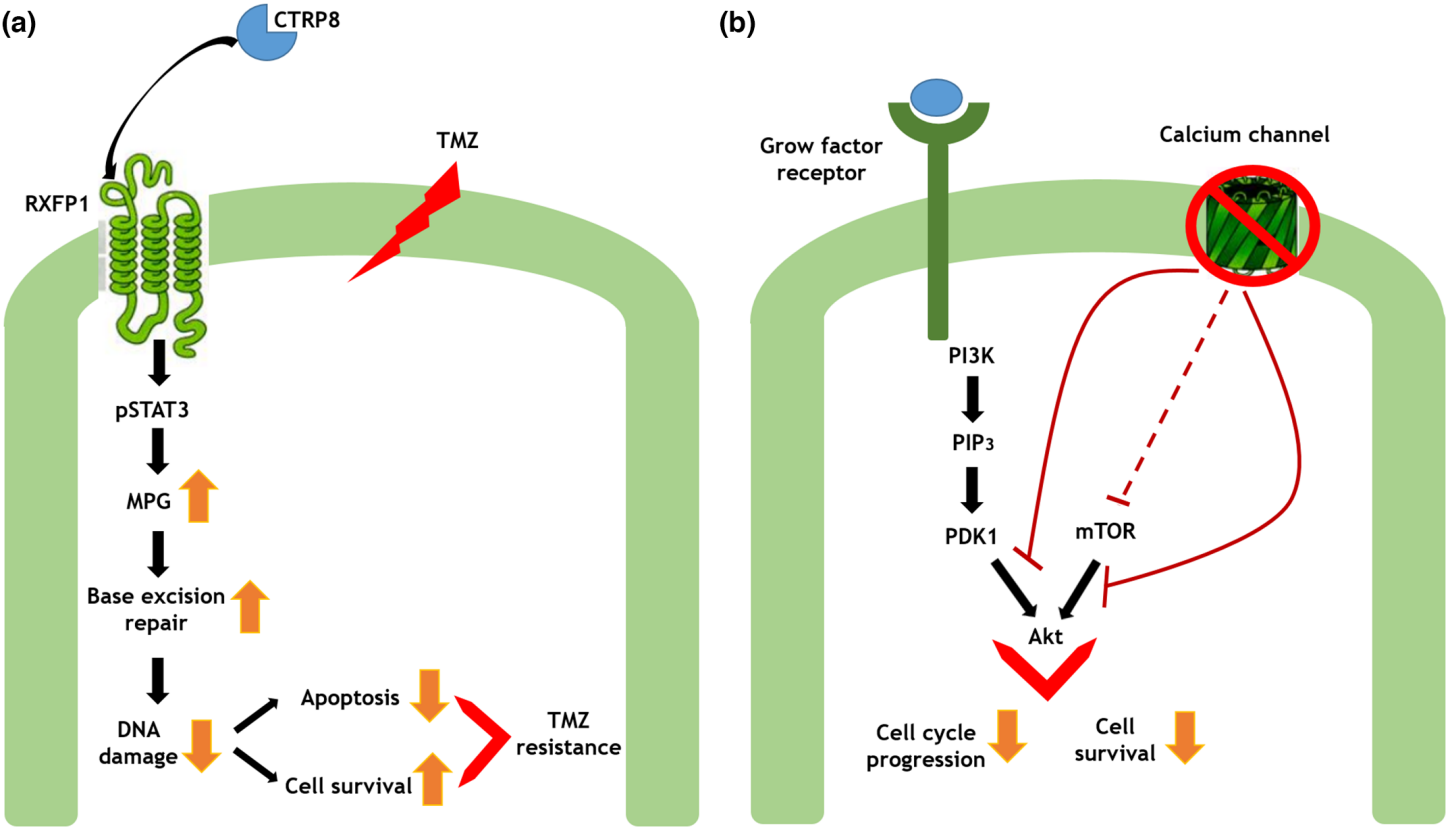
(a) Schematic representation of the role of CTRP8, encoded by C1QTNF8, in the development of resistance to temozolomide (TMZ) proposed by Thanasupawat et al. (2018). (b) Schematic representation of calcium channel inhibition and its role in glioblastoma.

The recent large‐scale genomic studies based on the Cancer Genome Atlas (TCGA) have been performed on the GBM TC, revealing a large number of mutations in tumor suppressor genes and oncogenes (Brennan et al., 2013; Lee et al., 2018). On the contrary, studies concerning the genomic profile of the PBZ are still few. Lemeè and colleagues performed array‐CGH analysis on PBZ biopsies from 10 patients. They reported large genomic alterations in four PBZ samples, also shared by cancer counterparts. However, the histological analysis revealed an infiltration of tumor cells on these PBZ samples (Lemée, Clavreul, Aubry, et al., 2015; Lemée, Clavreul, & Menei, 2015). In a study published by Mangiola and colleagues, several tumor‐associated alterations were present in brain adjacent to the tumor area, as well as in the TC: del(1p36), del(2p21), MDM2 and CDK4 amplification, amplification of 15q24.1, chromosome 19 and 22 losses. In contrast, they stated that genetic changes such as chromosome 7 gain, EGFR amplification, 6q27‐q29 region and 17p13 locus deletions were exclusive of the tumor tissue (Mangiola et al., 2013). Recently, we conducted a similar study on PBZ samples matched to the respective TC biopsies from 10 patients. Array‐CGH analysis on PBZ samples did not report any CNA in three cases, while genomic alterations were detected in seven samples, some of which matched with the corresponding tumor ones (Giambra et al., 2021). These alterations concerned imbalances already described in GBM, such as gain of chromosome 7 with EGFR amplification, deletion of chromosome 10 with PTEN loss, and loss of CDKN2A/2B locus on chromosome 9 (Aubry et al., 2016). By comparing the genomic profiles of the matched TCs and PBZs, we can classify these patients based on the strength of the genomic correlation. Interestingly, two patients with a very strong correlation relapsed or died within a year from diagnosis. The genomic correlation of two other patients was ranked as moderately strong, as the PBZ's CNA amount was lower and less represented than in TCs. One of these died of the disease within a year of being diagnosed and had the highest number of shared CNAs between TC and PBZ (Giambra et al., 2021). Furthermore, we focused on PBZ‐exclusive imbalances, identifying two gained regions, 11p11.2 and 16p13.3, containing genes of interest for GBM, which would be worth investigating in the future (EXT2, C1QTNF8, and CACNA1H) (Giambra et al., 2021). Particularly, EXT2 encodes an enzyme involved in the heparan sulfate (HP) biosynthetic system; HP is a component of the ECM and its deregulation in GBM promotes tumor invasiveness into the surrounding normal brain tissue (Ushakov et al., 2017). C1QTNF8 encodes for CTRP8, a ligand of the G protein‐coupled receptor RXFP1. CTRP8 promotes tumor motility and matrix invasion and its protecting role against TMZ has been demonstrated (Thanasupawat et al., 2018) (Figure 2a). CACNA1H encodes for the calcium voltage‐gated channel subunit alpha, whose inhibition decreases tumor proliferation and migration in GBM (Figure 2b) (Zhang et al., 2012).

### 4.2 Transcriptomic profiles

**FIGURE 3:**
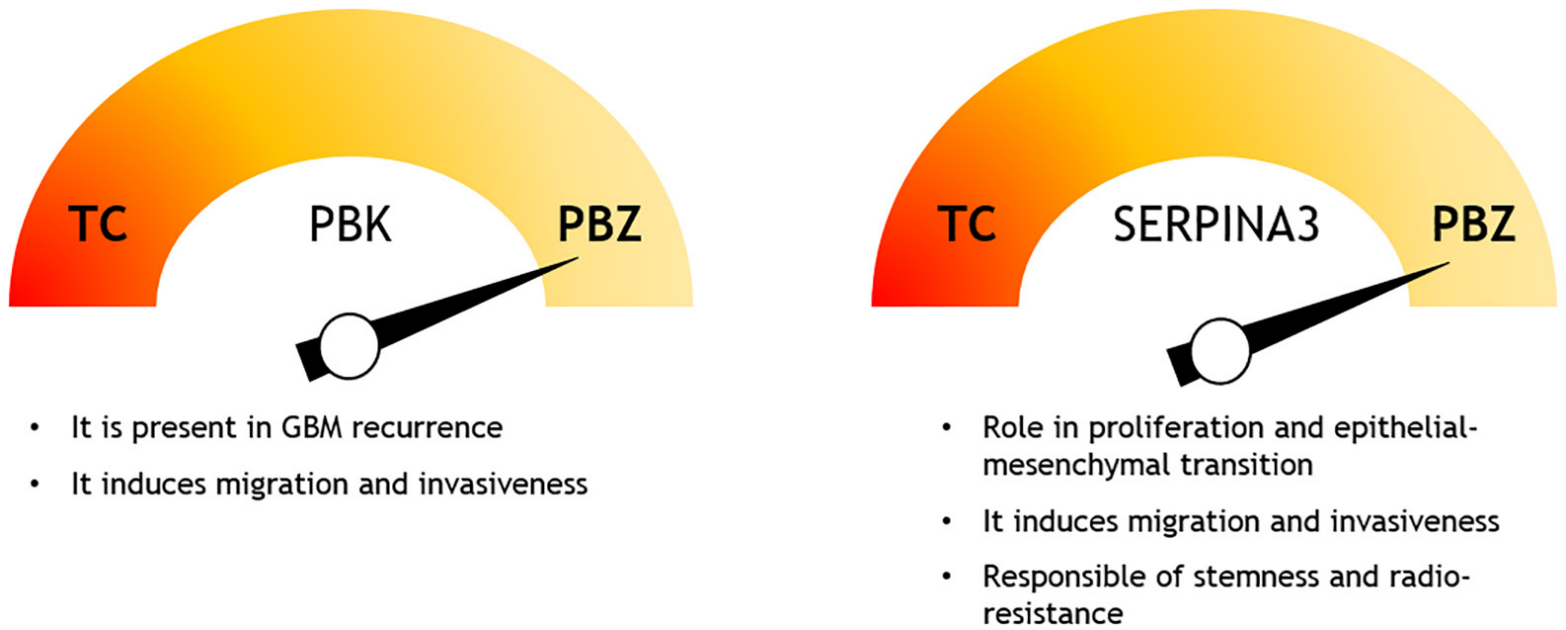
Higher expression levels of PBK (left) and SERPINA3 (right) in the peritumoral brain zone (PBZ) compared with the tumor core (TC) and summary of their function in glioblastoma (GBM).

PBZ shared with the TC some important features and this is confirmed by their partial matched transcriptomes. For instance, some notable genes upregulated both in TC and PBZ include VEGF and HIF1α, which regulate angiogenesis (Zhang et al., 2015), together with ECM‐associated genes like collagens and matrix metalloproteinases and microglial marker CD68 (Chen et al., 2014; Du et al., 2016). The expression of phosphorylated extracellular signal‐regulated kinases 1/2 (pERK1/2) and phosphorylated C‐jun NH2 terminal kinases (pJNK), both involved in the regulation of cellular senescence, and stem cell markers (such as Nestin, SOX2, Musashi, CD133, GD3, NG2, cMet) have been demonstrated in both TC and PBZ (Nimbalkar et al., 2021). Meanwhile, TC and PBZ top downregulated genes include SLC17A7 and CHD5, which are known as tumor suppressor genes in GBM and other systemic cancers, respectively (Baykara et al., 2017; Du et al., 2016; Lin et al., 2015; Ma et al., 2016). Among the differentially expressed genes in TC and PBZ, the top upregulated genes of PBZ include UBE2C, NUSAP1, IGFBP2, SERPINA3, and PBK, which are involved in cell growth and proliferation in several cancers (Han et al., 2014; Iyer et al., 2011; Joel et al., 2015; Wagner et al., 2004). Among these, IGFBP2 has been reported to have a dual status in PBZ: Hoelzinger et al. showed its downregulation through immunochemical analysis; conversely, Mangiola et al. described its upregulation through gene expression analysis on microarray (Hoelzinger et al., 2005; Mangiola et al., 2013). Upregulation of PBK and SERPINA3 is particularly interesting due to their functions. PBK, a protein related to the mitogen‐activated protein kinase (MAPKK) family, implicated in tumorigenesis of several systemic cancers including breast, prostate and lung cancers (Abe et al., 2000; Brown‐Clay et al., 2015; Park et al., 2006; Shih et al., 2012), is also upregulated in GBM, especially in GBM stem cells (Stangeland et al., 2015). PBK mRNA and protein expression is significantly upregulated in PBZ compared with TC. Furthermore, an increased expression of PBK in recurrent GBM is observed (Kruthika et al., 2019). From these observations, it could be assumed that infiltrating tumor cells moving to the normal neuroparenchyma from the tumor bulk have a higher PBK expression than non‐infiltrative ones. PBK overexpression likely helps them migrate and invade the PBZ, where they could contribute to tumor recurrence. Studies support the functional role of PBK in conferring tumor aggressiveness in GBM, showing that the treatment of glioma cells with PBK inhibitors reduces tumor volume in vivo (Joel et al., 2015; Sugimori et al., 2018) (Figure 3). Serpina3, a member of the serpin superfamily of protease inhibitors also known as α1‐antichymotrypsin (ACT) is highly expressed in the PBZ of GBM, having a role in proliferation, invasion, migration, EMT, stemness, and radio‐resistance. Its increased expression in recurrent GBM tissues and association with poor patient prognosis suggests that it contributes to the aggressive GBM phenotype (Nimbalkar et al., 2021) (Figure 3).

Other PBZ upregulated genes include those known to be associated with invasion and migration such as scinderin (SCIN) and stem cell markers such as aldehyde dehydrogenase 1 family member A1 (ALDH1A1) (Chen et al., 2014; Schäfer et al., 2012; Wang et al., 2014). In addition, genes related to neurogenesis like MAL, MOG, MAG, and MOBP are upregulated in PBZ, probably thanks to the native neuroparenchyma (Kruthika et al., 2019; Mangiola et al., 2013). Other upregulated genes such as KLK6, SH3GL3, and ENPP2, which are known to confer chemo‐resistance and promote invasion in GBM and other cancers (Drucker et al., 2013), pointed out to the aggressive nature of the infiltrating cells. Meanwhile, some important PBZ downregulated genes include NKAIN2 (tumor suppressor gene) and ULK2 (role in autophagy) (Shukla et al., 2014; Zhao et al., 2015). Taken together, gene expression analysis shows that cells in PBZ are likely undergoing active remodeling of the ECM, thus promoting cell invasion and migration. Meanwhile, TC is mostly enriched with genes and pathways related to tumor cell survival and growth, hypoxia and angiogenesis.

#### 4.2.1 miRNome profiles

In recent years, the role of non‐coding RNAs (ncRNAs) is emerging as major regulators of gene expression in both physiological and pathological conditions (Mousavi et al., 2022). Among ncRNAs, microRNAs (miRNAs) are ~20 ‐nucleotides long, endogenous, single stranded non‐coding RNA, which bind the 3′ untranslated region of target mRNAs, inhibiting mRNA stability or translation (Ahir et al., 2017; Friedman et al., 2009). The miRNome refers to the total of all expressed miRNAs. Valtorta and colleagues summarized angiogenesis‐related miRNAs in GBM, recognizing their role in tumor growth and potentially in invasiveness and capability of recurrence (Valtorta et al., 2020). Similarly, Chen and colleagues called “oncomiRs” miRNAs acting as tumor suppressors or tumor promoters in GBM (Chen et al., 2021). In Table 3 were reported the main known miRNAs associated with GBM.

**TABLE 3:** Summary of the main known microRNAs (miRNAs) involved in glioblastoma, their functions, and specific targets (Chen et al., 2021; Valtorta et al., 2020)

miRNA	Function	Target	Reference
miR‐7	Inhibits angiogenesis	EGFR, IRS‐1, IRS‐2, FAK, OGR	Valtorta et al. (2020)
miR‐15b	Inhibits angiogenesis	NRP‐2	
miR‐16	Inhibits angiogenesis	BMI‐1	
miR‐613	Inhibits angiogenesis	VEGFA	
miR‐29b, miR‐34a, miR‐101, miR‐137	Inhibits angiogenesis	STC1 induces eNOS, VEGF, and VEGFR2	
miR‐126/126‐3p	Inhibits angiogenesis	EGFL7	
miR‐124‐3p	Inhibits angiogenesis	NRP‐1/GIPC1 pathway	
miR‐129‐5p	Inhibits angiogenesis	WNT5a	
miR‐518b	Inhibits angiogenesis	PDGFRB	
miR‐520d‐5p	Inhibits angiogenesis	PTTG1	
miR‐296	Promotes angiogenesis	HGS	
miR‐93	Promotes angiogenesis	Integrin B8	
miR‐26a	Promotes angiogenesis	PTEN and PI3K/Akt	
miR‐9	Promotes angiogenesis	COL18A1, THBS2, PTCH1, PHD3, HIF‐1α/VEGF	
miR‐9‐5p, miR‐22‐3p, miR‐182‐5p	Promotes angiogenesis	RGS5, SOX7, and ABCB1	
miR‐378	Promotes angiogenesis	VEGFR2	
miR‐29b	Promotes angiogenesis	BCL2L2, which in turn regulates Ang‐2 and VEGF	
miR‐148a, miR‐31	Promotes angiogenesis	FIH1, HIF1α and Notch	Chen et al. (2021)
miR‐221/222	Inhibits apoptosis	PUMA	
miR‐335	Inhibits apoptosis	Bcl‐w	
miR‐218	Promotes apoptosis	CDK6, EGFR and ECOP	
miR‐195, miR‐455‐3p, and miR‐10a	Promotes temozolomide resistance	–	
miR‐181b and miR‐18c	Sensitizes to radio/chemotherapy	MGMT	
miR‐328	Sensitizes to mitoxantrone and doxorubicin	ABCG2	
miR‐29a, miR‐137	Inhibits GSCs self‐renewal	PDGF, RTVP‐1	
miR‐7	Tumor suppressor	FAK, EGFR, Akt, c‐KIT, TGFβ2, CDK6, AKT2, LRRC4, YBX1, CD24, and MTDH	
miR‐34a	Tumor suppressor	SIRT1, c‐Met, Notch1/2, PDGFRA, Msi1, Akt and Wnt	
miR‐128	Tumor suppressor	P70S6K1, SUZ12, BMI1, PDGFRα, EGFR, E2F3a, WEE1 and Msi1	
miR‐10b	oncomiR	HOXD10, uPAR, RhoC, PTEN, BCL2L11, TFAP2C, CDKN1A and CDKN2A	
miR‐21	oncomiR	HNRPK, TAp63, PDCD4, P53, TGF‐β, MMPs, Ras/Raf, ERK, ANP32A, SMARCA4, PTEN, SPRY2, and LRRFIP1	
miR‐93	oncomiR	Integrin b8	

In 2015, Piwecka and colleagues, for the first time, compared the global miRNA expression in adult invasive gliomas (mainly GBMs), the tumor margin, and non‐tumor brain tissues (Piwecka et al., 2015). They found 97 miRNAs differentially expressed between GBM and normal brain and 22 miRNAs differentially expressed between the tumor margin and normal brain; 21 out of the 22 were upregulated in the tumor as well. Meanwhile, Fazi and colleagues recognized some miRNAs overexpressed in the TC compared with the PBZ, among them miR‐21‐3p, miR‐196b‐5p, miR‐135b‐5p, and miR‐183–3p were already known as “oncomiRs” in GBM, whereas miR‐1246, miR‐1290, miR‐7641, and miR‐503‐5p had never been investigated in glioblastoma. By contrast, other miRNAs are enriched in the PBZ, such as miR‐219a, miR‐338‐3p, and miR‐338‐5p, playing a role in oligodendrocyte maturation, while miR‐34b and miR‐34c are known as tumor suppressor players (Fazi et al., 2015). More recently, Alfardus and coworkers highlighted distinct profiles between the TC and the PBZ. In particular, miR‐330‐5p and miR‐215‐5p were upregulated in the PBZ compared with the core, while miR‐619‐5p, miR‐4440, and miR‐4793‐3p were downregulated (Alfardus et al., 2021). In 2018, Hide and colleagues drew up a PBZ specific miRNA signature, composed by upregulated and downregulated miRNAs of the marginal area compared with the TC (miR‐219‐5p, miR‐219‐2‐3p, miR‐338‐3p, miR‐27b, miR‐23b and miR‐630, miR‐1246, miR‐642b, miR‐1181, miR‐H18, miR‐3195, miR‐3663‐3p, respectively) (Hide et al., 2018). Altieri et al. suggested a role of miRNAs during microglia polarization in the tumor margin that influences the aggressiveness of the disease (Altieri et al., 2021). miR‐627, identified in 2019, triggers the M2‐polarization (tumor promotion phenotype) of the microglia; while miR‐504 may allow M1 polarization (tumor suppression phenotype) (Bier et al., 2020; Li et al., 2019).

Recognizing PBZ‐specific miRNAs and their role will be important in developing miRNA‐based therapies or new diagnostic applications.

### 4.3 Proteomic profiles

In recent years, numerous proteomic studies have been reported in the field of glioma research (Com et al., 2012). The inter‐patient variability in the PBZ, similar to the one observed in the corresponding TC, complicates the identification of margin specific protein markers. For this reason, a large cohort of PBZ samples would be needed to identify the peculiar molecular signatures, but the constitution of a wide cohort raises the ethical issue of sampling normal brain tissue around the tumor. The identification of characteristic signatures is further complicated by the choice of an appropriate brain sample that can be used as a control (Lemée et al., 2013). Despite this, different studies demonstrated a gradient of protein overexpression from the core to the periphery of GBM, suggesting a dilution of the tumoral contingent from the core toward the margin (Com et al., 2012; Lemée et al., 2013, 2018; Lemée, Clavreul, Aubry, et al., 2015). Com et al. performed a protein analysis with the Isotope‐Coded Protein Label (ICPL) approach on three areas of GBM, including the IZ along with the TC and the PBZ, collected from five patients (Com et al., 2012). Specifically, authors focused their attention on a distinct subset of proteins, which had variable expression levels between the PBZ and the TC. Thirty‐one out of 259 proteins were overexpressed in the core compared with the margin in at least three out of five patients, and most of them formed a cohesive network whose core was composed mainly of β‐actin and two families of proteins: 14–3‐3 proteins and Heat Shock Proteins (Com et al., 2012). Interestingly, the 14–3‐3 zeta plays a key role in tumorigenesis because it is involved in cell growth, apoptosis prevention, and is related to astrocytoma malignancy (Hashemi et al., 2018). Lemèe and colleagues evidenced that upregulated proteins in PBZ are ubiquitous, such as components of basic cellular pathways, like DNA folding for histones (HIST1H2AC and HIST1H4A), or involved in the regulation of the osmotic pressure of blood, such as albumin. An upregulation of myelin basic protein and GFAP was also observed in the PBZ, which could be related to the high white matter content of this area. Nevertheless, proteins with an oncogenic role, such as crystallin B α‐chain (CRYAB) and the histone H3F3A, were also observed to be upregulated in the PBZ (Lemée et al., 2013). The same authors in 2018 performed a transcriptome and proteome integration analysis in TC and PBZ to identify related mRNA/protein levels in each specific area. The study showed a low correlation between the two omics profiles in TC and no correlation at all in PBZ (Lemée et al., 2018). Lama and coworkers demonstrated that activated extracellular signal‐regulated kinases 1 and 2 (ERK1/2) were shown to be present not only in the enhanced lesion but also in the tissue surrounding the neoplasia, both in reactive astrocytes and in apparently normal cells. In addition, the level of pERK1/2 expression did not differ significantly between the two areas (Lama et al., 2007). Mangiola and colleagues studied Nestin and JNK expression in the tumor and peritumoral area of GBM specifically. Nestin has been observed in most cells in the first, but rarely in the second area; moreover, total JNK (tJNK) was widely expressed, while phosphorylated JNK (pJNK), was found in a variable percentage of cells in both areas. According to this study, pJNK/nestin and (pJNK/tJNK)/nestin ratios in the PBZ seem to have some prognostic implications in GBM patients (Mangiola et al., 2007). Nestin overexpression, along with CD105, in the PBZ, has also been seen to be involved in the process of peritumoral angiogenesis (Sica et al., 2011).

## 5 TRANSLATIONAL ASPECTS

### 5.1 Magnetic resonance imaging of peritumoral brain zone

Several studies have shown the association between GBM preoperative magnetic resonance imaging (MRI) features and patients prognosis, but only a fraction of them have focused on imaging configuration of PBZ (Al‐Holou et al., 2020; Khalafallah et al., 2021; Molinaro et al., 2020). Moreover, the radiological identification of PBZ, intended as the normal brain tissue bordering the tumor, is problematic. Indeed, the TC boundary, which includes the necrotic area, can be easily identified by delineating the contrast enhancement area, visible on post‐gadolinium T1‐weighted (T1W) images. Conversely, the no‐enhancing regions, beyond these limits, may show radiological features that are not specific to tumor tissue and may be indistinguishable from vasogenic edema and other non‐tumor tissue alterations (D'Alessio et al., 2019; Kubben et al., 2012; Lemée, Clavreul, & Menei, 2015; Mangiola et al., 2013; Wang et al., 2015), characterized by T2‐weighted (T2W), fluid attenuated inversion recovery (FLAIR), peritumoral hyperintensity (Altieri et al., 2021; Grossmann et al., 2016; Ross et al., 2017). The latter usually fades toward the normal appearing brain tissue, making it very difficult to identify a clear‐cut border. Moreover, some studies on MRI‐guided biopsies report features of diffusively infiltrating gliomas intermixed with brain tissue apparently normal at MRI (Gill et al., 2014; Kubben et al., 2012; Sadeghi et al., 2008). Thus, most imaging studies define the PBZ as the whole area of T2W hyperintensity surrounding the CE T1W brain tissue identified as the border of the tumor (Lemée, Clavreul, & Menei, 2015). Tamura et al. explored the histopathology of this MRI‐defined PBZ (MR‐PBZ) and showed that a wide peritumoral T2W FLAIR hyperintensity is associated with elevated microvessel density and MIB‐1 index, while an arrow peritumoral FLAIR hyperintensity correlates with both low microvessel density and low MIB‐1 index (Tamura, Ohara, Sasaki, Morimoto, Kosugi, et al., 2018; Tamura, Ohara, Sasaki, Morimoto, Yoshida, et al., 2018). Moreover, Tamura et al. found that patients with high T2W FLAIR hyperintensity (beyond 1400 mm^3^) had more CD163+ cells in the TC (Tamura, Ohara, Sasaki, Morimoto, Yoshida, et al., 2018), which are involved in invasiveness, tumor cell migration, angiogenesis, immune evasion, edema formation, and worse survival rate (Bette et al., 2018). It must be noted that, to our knowledge, there are no other studies confirming Tamura's data. Moreover, a recent study found no association between the extent of T2W peritumoral hyperintensity and immune infiltration, suggesting that its size correlates with IDH mutation, being more pronounced for IDH‐wild type GBM, and is mostly related to vasogenic edema (Dubinski et al., 2021). These histopathological features are expected to correlate with some functional and microstructural modifications that may be explored with some MRI techniques. Vasogenic edema suggests increased vascular permeability (Chang et al., 2017; Hu et al., 2012), while enhanced vascular density may lead to increased blood volume and blood flow, and cellular infiltration can disrupt the structure of brain tissue. Multidirectional diffusion‐weighted imaging can be used to compute tensor‐based quantitative maps of water diffusion, such as apparent diffusion coefficient (ADC), fractional anisotropy, mean diffusivity, radial diffusivity, axial diffusivity, all of which may help distinguishing vasogenic edema from tumor infiltration and high‐cellularity regions (Beppu et al., 2005; Chen et al., 2013; Deng et al., 2010; Yan et al., 2019, 2020). Other MRI techniques, such as dynamic susceptibility contrast, arterial spin labelling, and T1W CE dynamic acquisitions, can be used to explore regional cerebral blood volume (CBV), cerebral blood flow, and vascular permeability indexes, all expected to be relatively higher in tumoral area associated with neoangiogenesis than in normal brain tissue (Bisdas et al., 2018; Han et al., 2018; Kickingereder et al., 2015; Min et al., 2013; Moon et al., 2012; Ryoo et al., 2013; Sadeghi et al., 2008; Sudre et al., 2020; Zhao et al., 2019). More recently, a relatively new functional MRI technique has been developed and allow to identify and quantify brain areas associated with glycolytic metabolism leading to lactate production and pH reduction, which can be identified using the MR signal modifications induced by chemical exchange saturation transfer (CEST) resulting in acidity maps (Akbari et al., 2021; Hagiwara et al., 2021). Considering the highly hypoxic metabolism of GBM, these MRI sequences may differentiate between IDH wild‐type and IDH‐mutant gliomas, which exhibit a different pH distribution gradient. Interestingly, a strong association between acidity maps and CBV was found also in T2W FLAIR peritumoral hyperintensities (Akbari et al., 2021; Wang et al., 2019). Apart from CEST imaging, all other techniques have entered the routine MRI protocols of primary brain tumors and are rapidly generating a huge amount of high‐definition structural and functional MRI multiparametric data, which can be correlated with histopathological, genetic, and molecular data. These type of analysis need high computing power and new imaging analysis methods. Radiomic is the emerging field related to computerized analysis of imaging multiparametric data (Gillies et al., 2016; Taha et al., 2021) and has already been applied to increase the ability to predict diagnosis, prognosis (Choi et al., 2019), survival stratification accuracy (Lao et al., 2017; Yang et al., 2021), site of tumor progression (Abrol et al., 2017), grade (Zacharaki et al., 2009), tumor's histological features (Chaddad et al., 2022), detection of genetic mutations and epigenetic alterations, that can affect clinical outcome and that can predict therapy response (Chaddad et al., 2022; Chang et al., 2017; Rathore et al., 2018). Applied to GBM, radiomic analysis proved to be effective in predicting IDH mutation, O6‐methylguanine‐DNA methyltransferase (MGMT) methylation and EGFRvIII mutation (Rathore et al., 2018; Vils et al., 2021), and 6‐month disease progression (Kim et al., 2019). Moreover, starting from categorized case series of patients with GBM in the Cancer Genome Atlas (TCGA) project, radiomic techniques for extrapolating predictive information on survival and pattern of tumor growth or recurrence aspects from MRI‐PBZ have emerged (Grossmann et al., 2016). Radiomic analysis coupled with biological knowledge on MRI‐PBZ will probably allow for a better delineation of the molecular features of MRI‐PBZ and a clearer description of regions at risk of tumor recurrence (Korfiatis et al., 2016; Li et al., 2018; Malik et al., 2021; Yan et al., 2020). CEST imaging is also being studied for routine use in patients with few reports available but promising results and useful applications in daily practice, especially where V‐ATPase inhibitory drugs will be used to treat high‐grade gliomas (HGGs) (Bertolini et al., 2019; Di Cristofori et al., 2015), and it is expected to further increase the multiparametric characterization of the MRI‐PBZ.

### 5.2 Complementary role of positron emission tomography in tumor margin definition

To better characterize the extent of diffuse glioma tissue infiltration, some recent articles investigated the capability of PET imaging with amino‐acid radiotracers, such as O‐(2‐[^18^F]‐fluoroethyl)‐L‐tyrosine (^18^F‐FET) and [^11^C]‐methionine (^11^C‐Met), to give complementary information to multiparametric MRI. In a prospective study, Dissaux et al. investigated tumor volume delineation by ^18^F‐FET PET and multiparametric perfusion MRI in 30 patients with newly diagnosed, untreated HGG. The authors demonstrated that multiparametric perfusion MRI volumes (rCBV, K2) were highly correlated with CE T1 gadolinium volumes whereas ^18^F‐FET PET provided complementary information, suggesting that the metabolically active tumor volume in patients with newly diagnosed untreated HGG is critically underestimated by contrast‐enhanced MRI (Dissaux et al., 2020). Furtak et al. performed a dual time‐point ^18^F‐FET‐PET/MRI at 10 (PET10) and 60 min (PET60) on two patients: the first with lesions with (T1‐CE) contrast enhancement and the second one with lesions without contrast enhancement (T1 and T2‐FLAIR). In both cases, areas with increased ^18^F‐FET were observed. In patient 1, there were multifocal areas of high ^18^F‐FET‐PET tracer uptake that were mismatched with the MRI findings. The histological diagnosis classified the first locus present in PET10 (not visible at T1‐CE) as a grade IV invasive glioma, while the histological diagnosis of the second location (correspondent to T1‐CE) was a WHO grade III lesion. The authors confirmed that the volume of high uptake in PET was greater than the volume of contrast enhancement in MRI. In addition, in patient 2 ^18^F‐FET uptake kinetics were noted and histology was diagnosed as a grade IV and a grade III (Furtak et al., 2021). Considering the high infiltrative growth pattern of gliomas, Sebok and colleagues characterized the extent of diffuse glioma tissue infiltration, beyond the visible lesion (i.e., beyond the T1‐CE lesion and/or T2/FLAIR‐defined tumor border), with ^18^F‐FET PET, and functional MRI CVR (blood oxygenation‐level‐dependent CVR [BOLD‐CVR]) mapping. Volumes of interest starting from the tumor lesion outward up to 30 mm were created for a detailed peritumoral PET and BOLD‐CVR tissue analysis. Authors detected hypermetabolism and impaired cerebrovascular reactivity beyond the standard MRI‐defined tumor border, suggesting active tumor infiltration in the peritumoral tissue. The hypermetabolism was still significantly present 6 mm beyond the visible glioma lesion, whereas significant BOLD‐CVR impairment was observed up to 12 mm (Sebök et al., 2021). Similarly, for identifying the imaging requirements for maximum resection of GBM with infiltrating GSCs, Inoue et al. divided ^11^C‐Met uptake area into five sub‐areas according to iso‐contour lines at tumor‐to‐contralateral normal brain tissue ratio (TNRs) of 1.2, 1.4, 1.6, 1.8, and ≥2.0. In all tumors, the ^11^C‐Met uptake volume at TNR of 1.4 was greater than the gadolinium (Gd)‐enhanced area on MRI. Immunohistochemistry revealed the existence of GSCs in the area showing ^11^C‐Met uptake at TNR 1.4 and no Gd‐enhancement (Inoue et al., 2021). All these studies demonstrated that GSCs and/or cancer cells could exist beyond the border of Gd‐enhanced tumor and the combination of PET imaging with multiparametric MRI could help to obtain maximum resection of GBM and to plan radiotherapy.

### 5.3 The concept of supramarginal resection and its impact on patients' prognosis

Nowadays it is clearly known that the OS of patients with GBM is significantly affected by the extent of CE tumor resection at brain MRI (Al‐Holou et al., 2020; Hervey‐Jumper & Berger, 2016; Li et al., 2016; Sanai et al., 2011). Less clear is how surgical resection of T2W FLAIR peritumoral hyperintense zone can affect prognosis of patients with GBM; although several authors support the idea that supramarginal resection can increase OS in patients with GBM (Molinaro et al., 2020; Pessina et al., 2017; Vivas‐Buitrago et al., 2022), while others do not (Jackson et al., 2020). The presumed benefit of resection of NE perilesional zone of GBM is questioned by the fact that supramarginal resection may be related to an increased risk of post‐operative neurological deficits (Molinaro et al., 2020) which may in turn adversely affect the OS of patients (approximately 6 months on median OS), abrogating all the benefits of resection (McGirt et al., 2009; Rahman et al., 2017). In a recent work published in 2020 by Molinaro and colleagues was showed that resection of NE peritumoral region increased median OS to 31 months in patients younger than 65 years of age (Molinaro et al., 2020). On the contrary, patients older than 65 years of age did not have any benefit from resection of NE perilesional region (Molinaro et al., 2020), despite some authors found contrary results (Vivas‐Buitrago et al., 2022). This huge increase of the median OS (almost 2 years on median OS), in contrast with the commonly reported median OS of 14–20 months, is counterbalanced by the increase risk of post‐operative neurological deficit in case of resection of the NE peritumoral region, although use of neurophysiological monitoring and pre‐operative diffusion tensor imaging should reduce this risk (Bello et al., 2008; Carrabba et al., 2016; De Witt Hamer et al., 2012; Di Cristofori et al., 2021; Raabe et al., 2014). One of the concerns of the resection of the FLAIR peritumoral hyperintensities is the amount of the region that need to be resected to increase the patient OS because of studies reporting contradictory results (Certo et al., 2019; Molinaro et al., 2020; Vivas‐Buitrago et al., 2022). In addition, some authors consider some anatomical locations more suitable for supramarginal resection than others (Khalafallah et al., 2021). Among authors supporting resection of NE area in GBMs, some suggest that NE region resection should be not more than 60%, whereas other suggest to resect as much as possible (Molinaro et al., 2020; Pessina et al., 2017; Vivas‐Buitrago et al., 2022). For example, Vivas‐Buitrago in 2021 (Vivas‐Buitrago et al., 2022) reported that resection of more than 60% of NE region do not affect OS and Li has explored this aspect in 2016 (Li et al., 2016) as well. Other research groups suggest resecting as much FLAIR hyperintense region as possible (Molinaro et al., 2020). These preliminary experiences on supramarginal resection could be influenced by the heterogeneity of the PBZ among patients and the lack of a single histological and molecular definition of the PBZ rather than the surgical technique used. In fact, as described previously, alterations seen at T2/FLAIR‐weighted images do not always describe the infiltrative cellularity that is the nourishment for tumor recurrence, but it may also be the expression of vasogenic edema (Min et al., 2013; Rathore et al., 2018). This consideration can justify contradictory findings. For example, while Schoeneggers et al. identified edema as an independent prognostic factor for poor outcome in GBM (Schoenegger et al., 2009), Lacroix et al., on the contrary, published their results including more than 400 GBM patients where the extent of peritumoral edema was not found to be an independent prognostic factor (Lacroix et al., 2001). Therefore, resection of FLAIR hyperintensities may lead to removal of vasogenic edema in some cases and may lead to infiltrative tumor resection in others. This fact can also justify the recurrences that are distant from the initial tumor site up to about 30% of cases (Pasqualetti et al., 2022). In this view, predicting the site of tumor recurrence would be detrimental to remove the FLAIR hyperintense areas that are the starting point for a tumor recurrence. In a work published in 2017, Chang et al. analyzed the surgical samples obtained from peritumoral margins of surgically resected gliomas. They were able to map the peritumoral NE region finding that tumor cellularity is inversely correlated with T2/FLAIR‐ and ADC‐weighted images and directly correlated with post‐contrast T1‐weighted images. These data confirmed the previous findings about MR diffusion and cellularity and guess that hyperintense T2/FLAIR‐weighted areas have a poor cellularity. With their model, they were able to map intratumoral heterogeneity and proposed a way to tailor a supramarginal resection (Chang et al., 2017). Tailoring a supramarginal resection can help reduce the risk of a post‐operative neurological deficit considering that the infiltrative peritumoral region of GBM is mixed with important white matter bundles that are not preserved by the NE part of the tumor. So far, the NE part of GBM may be unresectable unless there are permanent neurological deficits due to resection of functionally eloquent white matter bundles such as the cortico‐spinal tract. In this view understanding the biological behavior of the cellularity in the PBZ can lead to effective therapies that may be used to treat GBM in a multi‐modality way, especially when PBZ cannot be resected. Finally, in case of tumor recurrence, re‐irradiation of the supramarginal area represents a feasible local approach to be used as a stand‐alone therapeutic option or in addition to a new surgery. In detail, the available evidence suggests that re‐irradiation provides encouraging disease control and survival rates, and in some cases is related to a significantly reduced risk of death (Lo Greco et al., 2022).

## 6 CONCLUSION AND FUTURE PERSPECTIVES

In the near future, studies on the biological composition of the PBZ could have multiple translational impacts not only on the availability of target therapies capable of reducing the marginal parts of GBM, but could also play an important role in surgical planning and could change the way GBM is treated. Firstly, better knowledge and characterization of the PBZ may lead to biologically map the tumor on the brain MRI to guide the surgical resection to areas in which cellularity may be considered more aggressive or more chemo‐ or radio‐resistant. This approach may change the surgical way of treating GBM from a standard surgical procedure to a machine‐learning‐based procedure in which the tumor map built on models will guide the neurosurgeon. Such an approach, that is merely speculative, may increase the efficacy of surgery in obtaining a target cytoreduction with a decreased morbidity. Secondarily, a better knowledge of the PBZ and a planning obtained through a machine‐learning approach may concur to better select patients that will benefit from a supramarginal resection and those that will not.

## AUTHOR CONTRIBUTIONS

*Conceptualization*, A.B., C.G., and A.D.C.; *Resources*, M.G., A.D.C., S.V., R.M., V.B., and G.B.; *Writing – Original Draft*, M.G., S.V., R.M., and V.B.; *Writing – Review and Editing*, M.G. and A.B.; *Visualization*, M.G. and A.B.; *Supervision*, A.B., C.G., G.B., and R.M.

## CONFLICT OF INTEREST

The authors have no relevant financial or nonfinancial interests to disclose.

### PEER REVIEW

The peer review history for this article is available at https://publons.com/publon/10.1002/jnr.25134.
